# The effect of levosimendan on right ventricular function in patients with heart dysfunction: a systematic review and meta-analysis

**DOI:** 10.1038/s41598-021-03317-5

**Published:** 2021-12-16

**Authors:** Yaoshi Hu, Zhe Wei, Chaoyong Zhang, Chuanghong Lu, Zhiyu Zeng

**Affiliations:** 1 Department of Cardiology, The First Affiliated Hospital of Guangxi Medical University, Nanning, 530021 Guangxi China; 2 Guangxi Key Laboratory of Precision Medicine in Cardio-Cerebrovascular Diseases Control and Prevention, Guangxi Clinical Research Center for Cardio-Cerebrovascular Diseases, Nanning, 530021 Guangxi China

**Keywords:** Heart failure, Cardiomyopathies, Drug safety

## Abstract

Levosimendan exerts positive inotropic and vasodilatory effects. Currently, its effects on right heart function remain uncertain. This systematic review and meta-analysis is intended to illustrate the impacts of levosimendan on systolic function of the right heart in patients with heart dysfunction. We systematically searched electronic databases (PubMed, the Cochrane Library, Embase and Web of Science) up to November 30, 2020, and filtered eligible studies that reported the impacts of levosimendan on right heart function. Of these, only studies whose patients suffered from heart dysfunction or pulmonary hypertension were included. Additionally, patients were divided into two groups (given levosimendan or not) in the initial research. Then, RevMan5.3 was used to conduct further analysis. A total of 8 studies comprising 390 patients were included. The results showed that after 24 h of levosimendan, patients’ right ventricular fractional area change [3.17, 95% CI (2.03, 4.32), P < 0.00001], tricuspid annular plane systolic excursion [1.26, 95% CI (0.35, 2.16), P = 0.007] and tricuspid annular peak systolic velocity [0.86, 95% CI (0.41, 1.32), P = 0.0002] were significantly increased compared to the control group. And there is an increasing trend of cardiac output in levosimendan group [1.06, 95% CI (− 0.16, 2.29), P = 0.09 ] .Furthermore, patients’ systolic pulmonary arterial pressure [− 5.57, 95% CI (− 7.60, − 3.54), P < 0.00001] and mean pulmonary arterial pressure [− 1.01, 95% CI (− 1.64, − 0.37), P = 0.002] were both significantly decreased, whereas changes in pulmonary vascular resistance [− 55.88, 95% CI (− 206.57, 94.82), P = 0.47] were not significant. Our study shows that in patients with heart dysfunction, levosimendan improves systolic function of the right heart and decreases the pressure of the pulmonary artery.

## Introduction

Heart failure (HF) is a syndrome characterized by impaired ventricular filling and/or ejection function due many diseases, pulmonary and/or systemic congestion, and insufficient blood perfusion of organs and tissues. HF comprises heart dysfunction accompanied by clinical symptoms. HF has become a major cause of mortality and morbidity^1,2^. Compared to the left heart, the right heart has a limited influence on systemic diseases; thus, clinicians and researchers have not been very concerned about this facet in the past. However, poor right ventricular (RV) function has been confirmed as an independent prognostic marker for mortality in patients with heart dysfunction^3^. Right heart dysfunction develops for various reasons, primarily on of these three: (1) primary RV myocardial dysfunction, such as RV myocardial infarction due to coronary thrombosis and arrhythmogenic right ventricular cardiomyopathy (ARVC); (2) volume overload, such as right-sided valvular insufficiency and congenital heart diseases; and (3) pressure overload, including pulmonary hypertension, acute left ventricular (LV) failure and acute lung injury/hypoxia^4–6^. Right heart failure has a poor prognosis^7^. Furthermore, scholars^8^ have found that RV failure was associated with a high mortality rate, even exceeding LV failure, by observing the survival of patients with pulmonary arterial hypertension.

Catecholamines and phosphodiesterase inhibitors are often used in patients with acute decompensated HF, but the impact of these medications on prognosis is still controversial because they might lead to an increased risk of mortality. Under such circumstances, the Ca^2+^ sensitizer and K^+^ channel opener levosimendan is considered a safer choice of inotropic drug than other traditional drugs^9,10^. As a novel anti-HF drug, it plays an important role in enhancing myocardial contractility and distending systemic vasculature. In fact, it has been proven that levosimendan does indeed have the ability to improve left heart function in patients with heart dysfunction^11–13^. However, its influences on right ventricular systolic function and right ventricular load are not completely understood. Even though a number of published studies have reported the relationship between levosimendan and the right heart system, the small sample size in a single study, incomplete analysis parameters and different designed protocols have resulted in inconsistent conclusions. Therefore, a systematic review and meta-analysis is indispensable for synthesizing the evidence pertaining to the efficacy of levosimendan for RV function in patients with heart dysfunction by Doppler echocardiography and right heart catheterization.

## Material and methods

### Data sources and search strategy

We systematically searched pertinent original research documents from PubMed, The Cochrane Library, Embase, and Web of Science. The search time was from the time of each database’s inception to November 2020. Combinations of the following keywords were used to retrieve articles: “levosimendan”, “right heart or right ventricular (failure)”, “pulmonary hypertension”, “pulmonary embolism”, “perioperative” and “congenital heart disease”. The original research type was restricted to clinical controlled research published in English.

### Study selection criteria

Studies were included that met the following criteria: (1) the subjects of the studies were patients with heart dysfunction, and a majority of patients (≥ 70%) with heart function were classified as NYHA III/IV or patients with pulmonary hypertension (mPAP ≥ 25 mmHg before treatment); (2) in the treatment group, levosimendan was administered via intravenous infusion at a rate of 0.1–0.2 μg/kg/min for 24 h, with or without load; and the intervention measures for the control group were intravenous placebo, dopamine or prostaglandin; (3) observed endpoints included pulmonary artery pressure (PAP), pulmonary vascular resistance (PVR), tricuspid annulus plane systolic excursion (TAPSE), tricuspid annulus peak systolic velocity (S’), and right ventricular fraction area change (RVFAC) using Doppler echocardiography or right heart catheterization to assess the outcomes; and (4) the observation time was within 1 month after using levosimendan for the first time.

The exclusion criteria were as follows: (1) the subjects of the original studies were nonhuman; (2) the types of original studies were case reports, reviews, letters, comments, or conference abstracts; and (3) ongoing trials or literature with insufficient data from original studies.

### Data extraction

Two investigators (Yaoshi Hu and Zhe Wei) independently searched and screened the literature according to the inclusion and exclusion criteria and then extracted and verified the data. Disagreements were discussed in detail and resolved together with a third investigator (Chaoyong Zhang) if necessary or by contacting the original author. The following content was extracted from the included literature: (1) basic characteristics of the original studies, including first author, year of publication, study design, etc.; (2) sample size of the original studies; and (3) intervention strategies and study duration; (4) clinical end points and adverse events, etc.

### Quality evaluation

The quality of the included studies (randomized controlled trials, RCTs) was independently evaluated by two authors (Yaoshi Hu and Zhe Wei) using the Jadad scale, and any differences were resolved through discussion and adjudication. This method adopts the 0–5 score method, and trials scored one point for each area addressed. A score ≤ 2 was considered low-quality research, and ≥ 3 was considered high-quality research^14^. In addition, we conducted a quality assessment of cohort studies according to the Newcastle–Ottawa Scale (NOS), which primarily included selection, comparability and outcome evaluation. This method uses the 0–9 score scale, and studies with scores ≥ 6 are considered to be of high quality. However, the above quality assessment was not used as an exclusion criterion for eligible studies.

### Data synthesis and statistical analysis

For each study’s data, changes before and after treatment in the levosimendan and control groups were calculated. Then, we reported changes in the mean ± standard deviation (SD). Missing SD values were imputed based on the mean and SD of the changes reported in the original studies. According to the method mentioned in Chapter 16.1.3.2 of the Cochrane Handbook for Systematic 5.1.0 (Updated March 2011), a correlation coefficient (R) of 0.5 was adopted to calculate the standard deviation of the change. Subsequent meta-analysis was conducted using RevMan software (version 5.3; Cochrane Collaboration, Oxford, United Kingdom). The weighted mean difference (WMD) and 95% confidence interval (CI) were used to represent continuous data. If the 95% confidence interval does not overlap 0, the result is considered statistically significant. The Q test and I^2^ analysis were used to test heterogeneity among studies included in the pooled analysis. In the case of no significant difference (P > 0.1, I^2^ < 50%), a fixed-effects model was used for pooled analysis. Otherwise, we tried our best to identify possible sources of heterogeneity first, and then the random effects model was used for subsequent analysis (P ≤ 0.1, I^2^ ≥ 50%). Moreover, subgroup analysis or sensitivity analysis can be performed if necessary^15^.

## Results

### First item: results of the literature search

**Figure 1:**
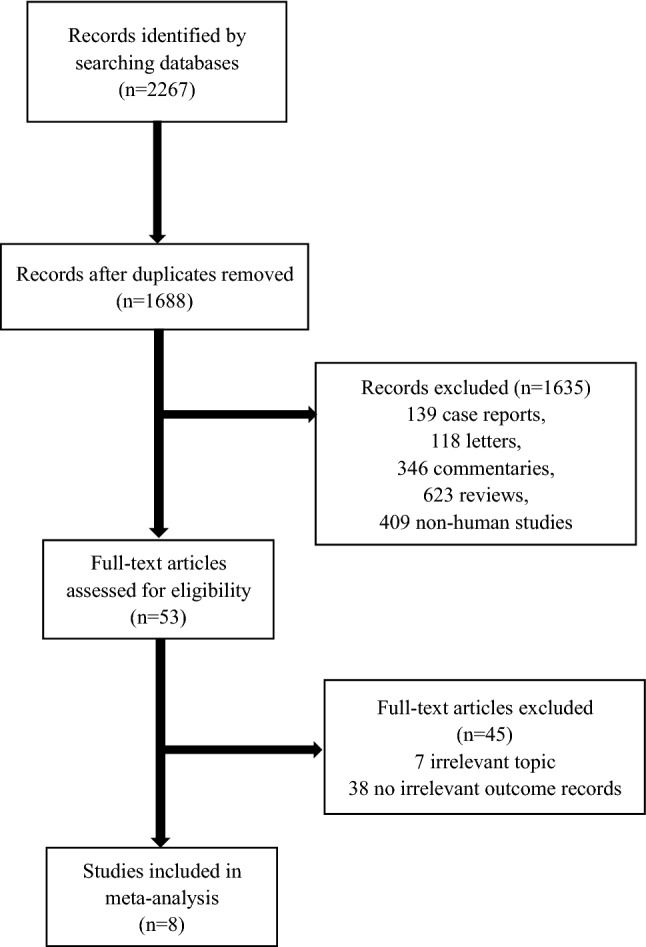
Flow chart for study selection.

As illustrated in Fig. 1, a total of 2267 studies were retrieved from the initial database search according to the study objectives and retrieval strategies. Among them, 933 were in the PubMed database, 69 were in the Cochrane Library database, 234 were in the Web of Science database and 1031 were in the Embase database. Of these, 2214 studies were removed due to being duplicates, animal studies, systematic reviews, letters, conference materials, and case reports. After full-text review, we excluded studies inconsistent with the topic of this study or without relevant data. In the end, 8 English studies were included^16–23^.

### Second item: study characteristics and quality assessment

There was a total of 8 original studies included, comprising 7 RCTs and 1 prospective cohort study. All literature was published from 2005 to 2019. The sample size was 390 cases in total, including 216 cases in the levosimendan group and 174 cases in the control group. Among trials reporting the study participants’ gender and age, there were 283 male patients (72.56%) and 107 female patients (27.44%). The mean ages of patients receiving levosimendan ranged from 49.6 to 69.9 years and from 45.7 to 72.6 years in the control group. All patients in the intervention group were administered levosimendan with or without a 10-min intravenous bolus infusion at 6–12 μg/kg, followed by a continuous 24-h infusion at 0.1–0.2 μg/kg/min, whereas patients in the control group started with infusion of placebo, dopamine or prostaglandin E1 for 24 h. Of these, 3 studies used levosimendan versus placebo, 2 studies used levosimendan versus dobutamine, 1 study used prostaglandin E1 in the control group, and patients in the control group did not receive any additional drug in the remaining 2 studies. Patient outcomes were assessed by Doppler echocardiography (4 RCTs and 1 prospective cohort study) or right cardiac catheterization (3 RCTs).

The trials varied in quality assessment from low to high. Three trials were classified as high quality (Jadad score ≥ 3 or NOS score ≥ 6), and 5 trials were classified as low quality (Jadad score = 2). More characteristics of the included articles are described in Table 1.

**Table 1:** Main characteristics and the quality assessment score (Jadad score) of the included studies in the meta-analysis.

Author	Year	No.of patients	Aetiology	Treatment	Control	Duration	Endpoints	Adverse events	Study design	Jadad score
Franz X. kleber	2009	28	PH	Levosimendan	placebo	24 h to 8 weeks	PVR, sPAP, mRAP, etc	Headache, Hypotension	RCT	4
Yan-Bo Wang	2019	59	ADHF	Levosimendan	placebo	24 h	TAPSE, sPAP, FAC, S’, etc	No description	RCT	2
Cristina Giannini	2017	54	FMR undergoing PMVR	Levosimendan	convention	24 h	TAPSE, sPAP, FAC, S’, etc	Relevant arrhythmia, surgery-related events	Cohort study	NOS: 6
Ozgur Ersoy	2013	20	High-risk valve surgery	Levosimendan	convention	24 h	PVR, mPAP, CO	Surgery-related events, side effect of the drugs	RCT	2
Mehmet Birhan Yilmaz	2009	40	Biventricular HF	Levosimendan	dobutamine	24 h	TAPSE, sPAP, FAC, S’, etc	No description	RCT	4
H. Duyu	2007	62	Ischaemic HF	Levosimendan	dobutamine	24 h	sPAP, S’, etc	No adverse events	RCT	2
John T. Parissis	2006	54	Advanced HF	Levosimendan	placebo	24 h	TAPSE, sPAP, S’, etc	No description	RCT	2
Deddo Moertl	2005	73	Decompensated CHF	Levosimendan	Prostaglandin E1	24 h	PVR, mPAP, CO	Hypotension, Diarrhea, Serum creatinine increase	RCT	2

*PH* percutaneous mitral valve repair, *FMR* functional mitral regurgitation, *HF* heart failure, *CHF* chronic heart failure, *mPAP* mean pulmonary artery pressure, *PVR* pulmonary vascular resistance, *sPAP* systolic pulmonary artery pressure, *FAC* right ventricular fractional area change, *TAPSE* tricuspid annular plane systolic excursion, *S’* tricuspid annular peak systolic velocity, *CO* cardiac output.

### Third item: efficacy

For each study, we calculated changes between pretreatment and posttreatment in both groups, reported as the mean ± SD. The meta-analysis results are shown as follows.

#### TAPSE

**Figure 2:**
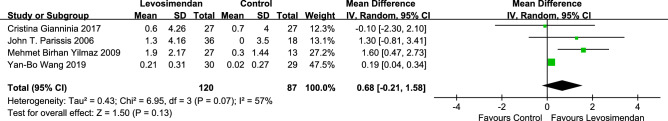
Forest plot for TAPSE-1.

**Figure 3:**
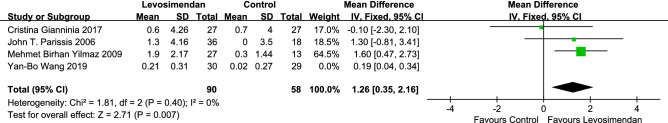
Forest plot for TAPSE-2.

For tricuspid annular plane systolic excursion (TAPSE), a total of 4 studies^19–21,23^ were included with 207 patients investigating changes in TAPSE after levosimendan or control treatment. These four studies showed an overall heterogeneity of I^2^ = 57% and P < 0.1, implying a moderate level of heterogeneity; hence, a random effects model was adopted. The results suggested that levosimendan did not significantly increase TAPSE in the overall analysis [WMD = 0.68 cm, 95% CI (− 0.21, 1.58), P = 0.13, Fig. 2]. Sensitivity analyses of the outcomes from the four studies implied that Yan’s data^20^ had a greater impact on the results compared to others’. We speculated that it was the cause of the moderate heterogeneity. After excluding the study, a total of 148 patients participated in the research. These three studies showed a low level of heterogeneity (I^2^ = 0%, P = 0.4), hence a fixed effects model was adopted. The results suggested that levosimendan significantly increased TAPSE in the overall analysis [WMD = 1.26 cm, 95% CI (0.35, 2.16), P = 0.007, Fig. 3].

#### RVFAC

**Figure 4:**
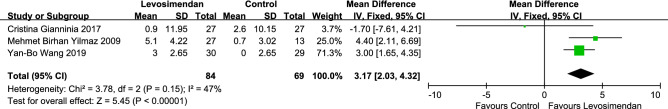
Forest plot for RVFAC.

For right ventricular fractional area change (RVFAC), 3 studies^20,21,23^ involving 153 patients investigated changes in RVFAC after levosimendan or control treatment. These three studies showed an overall heterogeneity of I^2^ = 47% and P = 0.15, implying a low level of heterogeneity among studies; hence, a fixed effects model was adopted. The results suggested that levosimendan significantly increased RVFAC levels in the overall analysis [WMD = 3.17%, 95% CI (2.03, 4.32), P < 0.00001, Fig. 4].

#### Tricuspid annular peak systolic velocity (S’)

**Figure 5:**
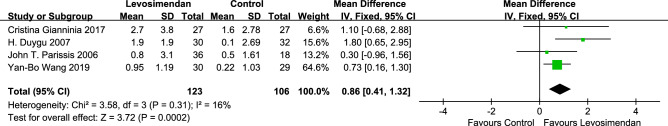
Forest plot for tricuspid annular peak systolic velocity.

For tricuspid annular peak systolic velocity (S’), 4 studies^18–21^ involving 229 patients investigated changes in S’ after levosimendan or control treatment. These four studies showed an overall heterogeneity of I^2^ = 16% and P = 0.31, implying a low level of heterogeneity among studies; hence, a fixed effects model was adopted. The results suggested that levosimendan significantly increased S’ in the overall analysis [WMD = 0.86 cm/s, 95% CI (0.41, 1.32), P = 0.0002, Fig. 5].

#### sPAP

**Figure 6:**
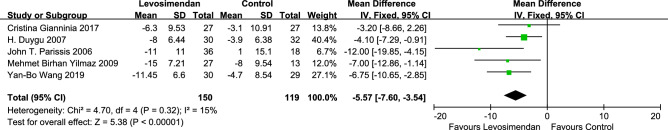
Forest plot for sPAP.

For systolic pulmonary artery pressure (sPAP), a total of 5 studies^18–21,23^ were included, involving 269 patients, investigating changes in sPAP after levosimendan or control treatment. These five studies showed an overall heterogeneity of I^2^ = 15% and P = 0.32, implying a low level of heterogeneity among studies; hence, a fixed effects model was adopted. The results suggested that levosimendan significantly decreased sPAP in the overall analysis [WMD = − 5.57 mmHg, 95% CI (− 7.60, − 3.54), P < 0.00001, Fig. 6].

#### mPAP

**Figure 7:**
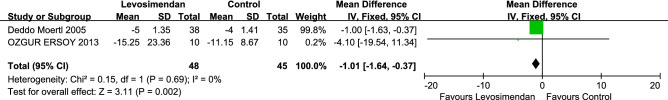
Forest plot for mPAP.

For mean pulmonary artery pressure (mPAP), 2 studies^17,22^ involving 93 patients investigated the changes in mPAP after levosimendan or control treatment. These two studies showed an overall heterogeneity of I^2^ = 0% and P = 0.69, implying a low level of heterogeneity among studies; hence, a fixed effects model was adopted. The results suggested that levosimendan significantly decreased mPAP in the overall analysis [WMD = − 1.01 mmHg, 95% CI (− 1.64, − 0.37), P = 0.002, Fig. 7].

#### PVR

**Figure 8:**
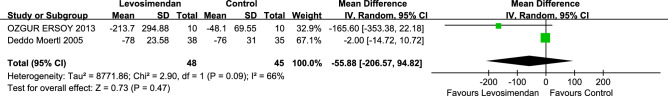
Forest plot for PVR.

For pulmonary vascular resistance (PVR), 2 studies^17,22^ involving 93 patients investigated changes in PVR after levosimendan or control treatment. These two studies showed an overall heterogeneity of I^2^ = 66% and P = 0.09, implying a moderate level of heterogeneity among studies; hence, a random effects model was adopted. For this analysis, the results suggested that levosimendan did not significantly reduce PVR in the overall analysis [WMD = − 55.88, 95% CI (− 206.57, 94.82), P = 0.47, Fig. 8].

#### CO

**Figure 9:**
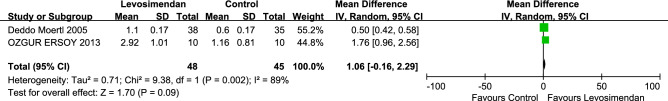
Forest plot for CO.

For cardiac output (CO), 2 studies^17,22^ involving 93 patients investigated changes in PVR after levosimendan or control treatment. These two studies showed an overall heterogeneity of I^2^ = 89% and P = 0.002, implying a high level of heterogeneity among studies; hence, a random effects model was adopted. For this analysis, the results suggested that levosimendan did not significantly increase CO in the overall analysis [WMD = 1.06, 95% CI (− 0.16, 2.29), P = 0.09, Fig. 9].

### Fourth item: safety

Among the 8 studies included, safety issues were reported in 5 studies^16–18,21,22^, for which we performed a descriptive analysis.

Two studies^17,21^ reported a number of incidents related to surgical safety, such as operation time, length of stay in the ICU, number of IABP implants, and mortality, but the results showed no significant difference between the levosimendan and control groups. Another study^18^ reported that no adverse events occurred in either group. To the best of our knowledge, the most common adverse reactions to levosimendan are hypotension, headache, and ventricular tachycardia. However, these adverse reactions were rarely observed in the included studies. Although Yanbo Wang^20^ mentioned in his study that systolic and diastolic blood pressure within 24 h after medication exhibited a downward trend compared to before medication, overall blood pressure levels were still within the normal range. Moreover, there was no significant difference between the two groups. According to Cristina Giannini’s study^21^, there were no reports of any related arrhythmia during patient hospitalization. Only 3 cases of new onset atrial fibrillation occurred, one in the control group and two in the levosimendan group, but there was no significant difference between the groups. Kleber. F. X^16^ reported the incidence of various adverse events in response to levosimendan in detail. In his study, a total of 19 patients (65 adverse events) were recorded, of which 11 patients (61%) and 8 patients (80%) were in the levosimendan group and control groups, respectively. Four patients (8 episodes) in the levosimendan group developed hypotension, resulting in dose reduction in three patients, while no hypotension occurred in the control group. Headache was reported by 4 patients (levosimendan, n = 3; placebo, n = 1). The proportions of drug-related or serious adverse events in the levosimendan (33%) group and the control (30%) group were parallel. Deddo Moertl^22^ also reported adverse events of hypertension. In the levosimendan group, 12 patients (34%) were administered a reduced dose of medicine due to hypotension. Moreover, during the 24-h infusion, levosimendan had to be withdrawn in 3 patients, which was attributed to serious hypotension and elevated serum creatinine.

## Discussion

To the best of our knowledge, this is the first systematic review and meta-analysis that comprehensively illustrates the efficacy of levosimendan for RV function in patients with heart dysfunction, including pulmonary artery pressure, pulmonary vascular resistance, tricuspid annular plane systolic excursion, tricuspid annular peak systolic velocity, right ventricular fractional area change and cardiac output. Although the previous meta-analysis^24^ also compared the RV function of the two groups to a certain degree, it emphasized on rather the comparison of the LV systolic function than the RV. And there were just small sample sizes, incomplete analysis parameters, and different analysis methods and proposals in some other researches, having made it difficult to compared results. As such, we conducted this study. In this study, the comparison of RVFAC and S’ between the two groups were added to replace the LVEF in the previous. Moreover, this study included more initial studies on the comparison between two groups, which made it more convincing. Our meta-analysis included a total of 8 studies with a combined study population of 390 patients and provides evidence that levosimendan decreases both the mPAP and sPAP in patients with heart dysfunction. Meanwhile, the drug also increased levels of RVFAC and TAPSE, as well as tricuspid annular peak systolic velocity. And there is an increasing trend of cardiac output in levosimendan group. However, we failed to offer evidence that levosimendan reduces PVR.

There was moderate heterogeneity in the current meta-analysis among studies in the analysis of TAPSE (57%), and the results suggested that levosimendan did not significantly increase TAPSE in the overall analysis. Sensitivity analyses of the outcomes from the four studies implied that Yan’s data^20^ had a greater impact on the results compared to others’. We reviewed the original literature once again and found that their measurement method was similar to that reported in other researches, but the results of measured TAPSE were much lower than the normal value or even the value of others, which led to the change value (mean ± standard deviation) of TAPSE being smaller. What’s more, there were no significant differences in the sample size or baseline characteristics of patients included (such as age, NYHA class, left ventricular ejection fraction, and duration of medication, etc.) between Yan's research and others’, which was believed not to lead to such a remarkable difference. Considering from the perspective of clinical heterogeneity, we speculated the existence of measurement or recording errors in Yan’s study, and tended that it was the cause of moderate heterogeneity. We tried to contact the author via email for further confirmation but failed. After excluding this study, the remaining three studies showed a low level of heterogeneity (I^2^ = 0%), and the results suggested that levosimendan significantly increased TAPSE in the overall analysis. In addition, there were also moderate levels of heterogeneity among studies in the analysis of PVR (I^2^ = 66%) and CO (I^2^ = 89%). Regrettably, the included studies were too few to perform sensitivity analysis to explore the source of heterogeneity. Therefore, we used the random effects model and found that levosimendan did not reduce PVR or increase CO in such patients. For FAC, sPAP, mPAP and tricuspid annular peak systolic velocity, the results were regarded as reasonably reliable due to the low heterogeneity. Although the results of mPAP and PVR were inconsistent with the previous analysis^24^, in the view of the lower heterogeneity (I^2^: 0% vs. 92%), primitive data types as well as the rationality of statistical methods, it could be thought that the results of the current study were more reliable to a certain extent.

Assessment of RV function is more challenging, and the relevant parameters are harder to establish than those of the left ventricle as a result of the irregular and complex geometric morphology of the RV^25^. At present, echocardiography is the most commonly used method in clinical practice for evaluating RV function. RVFAC and TAPSE are typically regarded as symbols of the longitudinal function of the RV. According to the 2010 ASE guidelines, it is easy to obtain the value of TAPSE from echocardiography, which can be used as a routine measurement index of RV function. Decreased TAPSE (< 16 mm) is associated with weakened RV systolic function^26^. Moreover, technicians are also able to evaluate RV function by calculating the value of RVFAC via a related formula. RV systolic dysfunction is considered when RVFAC < 35%^26^. In addition, the tricuspid annular peak systolic velocity (S’) can be harvested quickly and repeatedly at the same time. If the value of S’ is slower than 11.5 cm/s, it predicts an RV ejection fraction less than 45% with a sensitivity of 90% and a specificity of 85%^27^. Similarly, in regard to PAH patients, De Castro, S. reported in his article that a value of S' less than 9.5 cm/s was the optimal value for predicting RVEF < 40%^28^. On the other hand, right heart catheterization is also a major and widely used measurement to estimate RV load, which provides key information, such as PVR, sPAP, mPAP, CO, etc.

In patients with heart dysfunction, even systemic diseases, RV failure usually accounts for the leading cause of mortality. Right heart failure has a much worse prognosis than left heart failure^7^. Over time, people have paid increasing attention to the right heart, from anatomy to pathophysiology to function. The geometric morphology of the RV is complex and irregular and is composed of the inflow tract (tricuspid valve, chordae, and papillary muscle), trabecular apex myocardium and funnel part. Systemic venous blood return is received by the right atrium, passes through the tricuspid valve to the RV and is finally pumped into the pulmonary circulation through the pulmonary valve. The pressure of pulmonary circulation is much lower than that of systemic circulation, so the wall of the RV is much thinner than that of the LV. As pulmonary vascular resistance and pulmonary arterial pressure increase, the myocardial contractility of the RV will also be enhanced up to four- to fivefold to maintain stroke volume^29^. This compensatory mechanism is called the "coupling" of the ventricle and its load, which is achieved primarily by myocardial hypertrophy. As the disease progresses, cardiac output is maintained by relying on RV cavity diastole and increased heart rate^6^. Then, RV decompensates, and "decoupling" occurs^30^. Eventually, it comes into RV dysfunction, even right heart failure. As mentioned above, the causes of right heart failure vary; hence, the management of right heart failure should aim at the pathogenic factors of the primary disease. Additionally, it is a key step to follow a three-pronged approach: enhancing RV myocardial contractility, decreasing afterload, and optimizing volume load^31^.

Levosimendan, described as a novel anti-heart failure agent, has two major pharmacological mechanisms. It exerts a positive inotropic effect by binding to cardiac troponin C and enhancing the sensitivity of cardiac muscle to calcium; additionally, it exerts a vasodilator effect by mediating the opening of ATP-dependent potassium channels in smooth muscle cells of blood vessels (primarily in the pulmonary arterial vascular system)^9,32,33^. At this point, levosimendan could be used not only as an inotrope but also as an inodilator in clinical practice. Moreover, levosimendan has several advantages over traditional inotropes: (1) the positive effect of contractility is not related to the increasing concentration of intracellular calcium, so myocardial oxygen consumption will not increase; (2) if beta-blockers are given, the effect is not compromised; (3) the action is prolonged by forming active metabolites; and (4) the agent has a beneficial effect on peripheral organ (kidney and liver) perfusion and function via vasodilation^9,34,35^. Furthermore, it has been confirmed that levosimendan subserves heart failure in some practical studies. For instance, repeated intravenous levosimendan benefited patients with advanced heart failure by reducing hospitalization and mortality rates^36–38^. A meta-analysis comprising 1065 patients with acute heart failure/cardiogenic shock revealed that levosimendan-treated patients with acute coronary syndrome appeared to have haemodynamic benefit but did not identify an adverse effect on worsening heart failure or mortality^39^.

Our pooled analysis showed that levosimendan improved RV systolic function for enhancing RVFAC and TAPSE, as well as tricuspid annular peak systolic velocity. Actually, some previous studies have also shown that patients' right heart function could be improved after levosimendan. Asli Kurtar Mansiroglu et al.^40^ identified an increase in RVEF and LVEF, as well as an improved RV diastolic index, in 47 patients with acute compensatory heart failure. Additionally, they observed the recovery of neurohormones and alleviation of symptoms, manifested as a significant decrease in serum NT-pro BNP levels and NYHA class. In the study performed by Fatma Alibaz-Oner et al.^41^, the impact of levosimendan on RV systolic and diastolic parameters, isovolumic myocardial acceleration (IVA) and peak myocardial velocity during isovolumic contraction (IVV) derived from tissue Doppler imaging was evaluated in 30 patients with ischaemic heart failure. Their data suggested a favourable effect of levosimendan therapy on RV systolic function due to the significant increase in IVA and IVV.

Levosimendan improves RV systolic function through a variety of mechanisms. First, due to the positive inotropic effect, levosimendan increases LVEF and LV filling pressures, which might exert favourable effects on RV systolic function^19,42^. Additionally, pulmonary arterial pressure decreases in part as a result of the amelioration of LV function. Certainly, the agent could also enhance RV contractility similar to that of the LV. Leppikangas et al.^43^ also reported that after continuous infusion of Levosimendan for 24 h, cardiac index (CI) and stroke volume index (SI) were significantly enhanced. As mentioned in our results, levosimendan could enhance RV contractility. It is all known that stroke volume (SV) is affected by preload, myocardial contractility, and afterload. And the regulation of the pumping function of the heart by changing the myocardial contractility, independent of the other two, is called homometric regulation. In the case of a constant heart rate, CO is positively related to SV. Accordingly, SV and even CO should be theoretically increased, as a result of enhancement of RV systolic function. Intriguingly, the consistent conclusion was not observed in our pooled analysis. There were two possible explanations for this outcome. One is that levosimendan also exerts vasodilatory effects, leading to the reduction of preload, which cause a similar consequent on SV and CO based on heterometric autoregulation. The other is the statistical limitations for small sample size aggregated or the original research. Second, levosimendan exerts vasodilating effects on the pulmonary arterial system, resulting in a reduction in PVR and PAP^44^. Therefore, the RV afterload is reduced, and the mechanical efficiency is improved. Previously, it was reported that PVR was reduced after levosimendan by Slawski et al.^45^. However, our pooled analysis demonstrated that there was only a reduction in PAP but not PVR. Of note, the difference in the dose of levosimendan used might be a possible reason for these differences. Undeniably, we included only a few studies in the analysis of PVR, which weakens the persuasiveness of the results. Third, it is believed that levosimendan improves ventricular-vascular coupling of the RV, combining the dual benefits of pulmonary vasodilatation and positive inotropy^20,23^.

Undoubtedly, our research has several limitations. First, our study’s data were extracted from other studies. Due to the inherent limitations of these studies, such as heterogeneity with respect to data selection, our pooled results might have produced some biases towards positive results. Second, high heterogeneity of PVR was observed, and the sources of this heterogeneity remain unknown and are attribute to the few numbers of included studies. The low number of clinical studies, small samples of each included study, and low-quality trials weaken the conviction of the results to a certain extent. Third, although our study showed that levosimendan was beneficial for RV function in some respect, the follow-up time was too short to verify the long-term efficacy of the drug. Forth, the most common etiology of patients enrolled are heart failure (mitral regurgitation, ischemia, etc.), but we failed to obtain specific information of these patients. Thus, it limits us to perform further subgroup analysis. However, the results might suggest that levosimendan is effective for RV failure caused by all kinds of heart failure. Surely, further large-scale, multicentre strict RCTs and more pertinent evaluation parameters (three-dimensional right ventricular ejection fraction, right ventricular myocardial strain, 6-min walk test, etc.) are required to verify the positive effects of this drug.

## Conclusions

Our meta-analysis provides evidence that levosimendan improves the systolic function of the right heart and decreases the pressure of the pulmonary artery in patients with heart dysfunction. However, the current meta-analysis was based on observational studies. Due to the lack of other evaluation parameters, these conclusions should be interpreted with caution.

## Data Availability

All data generated or analysed during this study are included in this published article (and its Supplementary Information files).
